# Protein subcellular localization prediction of eukaryotes using a knowledge-based approach

**DOI:** 10.1186/1471-2105-10-S15-S8

**Published:** 2009-12-03

**Authors:** Hsin-Nan Lin, Ching-Tai Chen, Ting-Yi Sung, Shinn-Ying Ho, Wen-Lian Hsu

**Affiliations:** 1 Bioinformatics Program, Taiwan International Graduate Program, Academia Sinica, Taipei, Taiwan, Republic of China; 2 Bioinformatics Lab., Institute of Information Science, Academia Sinica, Taipei, Taiwan, Republic of China; 3 Institute of Bioinformatics, National Chiao Tung University, Hsinchu, Taiwan, Republic of China

## Abstract

**Background:**

The study of protein subcellular localization (PSL) is important for elucidating protein functions involved in various cellular processes. However, determining the localization sites of a protein through wet-lab experiments can be time-consuming and labor-intensive. Thus, computational approaches become highly desirable. Most of the PSL prediction systems are established for single-localized proteins. However, a significant number of eukaryotic proteins are known to be localized into multiple subcellular organelles. Many studies have shown that proteins may simultaneously locate or move between different cellular compartments and be involved in different biological processes with different roles.

**Results:**

In this study, we propose a knowledge based method, called KnowPred_site_, to predict the localization site(s) of both single-localized and multi-localized proteins. Based on the local similarity, we can identify the "related sequences" for prediction. We construct a knowledge base to record the possible sequence variations for protein sequences. When predicting the localization annotation of a query protein, we search against the knowledge base and used a scoring mechanism to determine the predicted sites. We downloaded the dataset from ngLOC, which consisted of ten distinct subcellular organelles from 1923 species, and performed ten-fold cross validation experiments to evaluate KnowPred_site_'s performance. The experiment results show that KnowPred_site _achieves higher prediction accuracy than ngLOC and Blast-hit method. For single-localized proteins, the overall accuracy of KnowPred_site _is 91.7%. For multi-localized proteins, the overall accuracy of KnowPred_site _is 72.1%, which is significantly higher than that of ngLOC by 12.4%. Notably, half of the proteins in the dataset that cannot find any Blast hit sequence above a specified threshold can still be correctly predicted by KnowPred_site_.

**Conclusion:**

KnowPred_site _demonstrates the power of identifying related sequences in the knowledge base. The experiment results show that even though the sequence similarity is low, the local similarity is effective for prediction. Experiment results show that KnowPred_site _is a highly accurate prediction method for both single- and multi-localized proteins. It is worth-mentioning the prediction process of KnowPred_site _is transparent and biologically interpretable and it shows a set of template sequences to generate the prediction result. The KnowPred_site _prediction server is available at http://bio-cluster.iis.sinica.edu.tw/kbloc/.

## Background

Protein subcellular localization (PSL) is important to elucidate protein functions as proteins cooperate towards a common function in the same subcellular compartment [1]. It is also essential to annotate genomes, to design proteomics experiments, and to identify potential diagnostic, drug and vaccine targets [2]. Determining the localization sites of a protein through experiments can be time-consuming and labor-intensive. With the large number of sequences that continue to emerge from the genome sequencing projects, computational methods for protein subcellular localization at a proteome scale become increasingly important.

Most existing PSL predictors are based on machine learning algorithms. They can be categorized by the feature sets used for building prediction models. A group of methods use features derived from primary sequence [3-7]; some utilize various biological features extracted from literature or public databases [2,8-13]. Other features are also used in different methods, e.g., phylogenetic profiling [14], domain projection [15], sequence homology [5], and compartment-specific features [16].

A simple and reliable way to predict localization site is to inherit subcellular localization from homologous proteins. Therefore, in [5] a hybrid method was proposed, which combined an SVM based method with a sequence comparison tool to find homology to improve the performance. However, some homologous proteins are not similar in sequences, but in structures. For example, the sequence identity between proteins *1aab *and *1j46 *is only 16.7% but they are structurally homologous and classified into the same family (*HMG-box*) in the SCOP classification. For such cases, it is difficult to discover the homologous relationship using sequence comparison methods. Profile-profile alignment methods [17-21] are capable of identifying remote homology; nevertheless, they are relatively slow.

Most of the PSL prediction systems are established particularly for single-localized proteins. A significant number of eukaryotic proteins are, however, known to be localized into multiple subcellular organelles [22,23]. In fact, proteins may simultaneously locate or move between different cellular compartments and be involved in different biological processes with different roles. This type of proteins may take a high proportion, even more than 35% [22]. In addition, the majority of existing computational methods have the following disadvantages [23]: 1) they only predict a limited number of locations; 2) they are limited to subsets of proteomes which contain signal peptide sequences or with prior structural/functional information; 3) the datasets used for training are for specific species, which is not sufficiently robust to represent the entire proteomes. Thus, most of the computational methods are not sufficient for proteome-wide prediction of PSL across various species.

Thus in this study, we propose a knowledge based approach, called KnowPred_site_, using local sequence similarity to find useful proteins as templates for site prediction of the query protein. It is designed to predict localization site(s) of single- and multi-localized proteins and is applicable to proteome-wide prediction. Furthermore, it only requires protein sequence information and no functional or structural information is required. Notably, prediction results can be explained by the template proteins which are used to vote for the localization sites. The Knowledge-based prediction scheme has been shown to be effective in predicting protein secondary structure [24,25] and local structure [26]. To evaluate our knowledge-based site prediction method, we used the ngLOC dataset [23] to perform ten-fold cross validation to compare with existing methods. The dataset consists of ten subcellular proteomes from 1923 species with single- and multi-localized proteins. KnowPred_site _achieved 91.7% accuracy for single-localized proteins and 72.1% accuracy with both sites correctly predicted for multiple localized proteins.

## Methods

### The main idea behind KnowPred_site_

**Figure 1:**
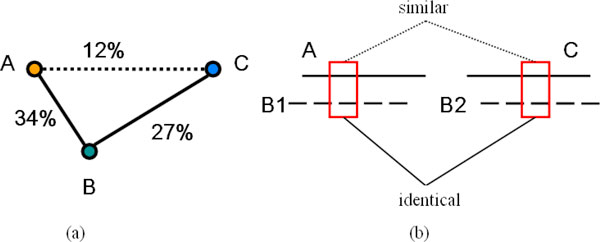
**Two different transitivity relationships**. (a) Protein *A *and protein *B *share sequence identity of 34%, and protein *B *and protein *C *share sequence identity of 27%, whereas protein *A *and protein *C *only share sequence identity of 12%. We infer the homologous relationship between *A *and protein *C *through protein *B*. (b) Protein *A *and protein *C *are aligned with protein *B1 *and protein *B2*. The peptide fragments of *B1 *and *B2 *besieged by the rectangles are identical, the two corresponding peptide fragments of *A *and *C *are considered to be similar.

KnowPred_site _predicts PSL based on a knowledge base, which is constructed to capture local sequence similarity of two proteins even when they have sequence identity less than 25%. However, such local similarity is difficult to be detected using the traditional alignment algorithm due to the low sequence similarity. Therefore we adopt the transitivity relationship, which was firstly used in [27] for clustering protein sequences, to capture local similarity between protein sequences. Transitivity refers to deducing a possible similarity between protein *A *and protein *C *from the existence of a third protein *B*, such that *A *and *B *as well as *B *and *C *are homologues if the sequence identity between *A *and *B *as well as that between *B *and *C *is above the predefined threshold. Figure 1(a) shows an example of transitivity relationship among protein *A*, protein *B*, and protein *C*. Protein *A *and protein *B *share sequence identity of 34%, and protein *B *and protein *C *share sequence identity of 27%, whereas protein *A *and protein *C *only share sequence identity of 12%. Using the transitivity relationship, remote homologous relationship and local similarity between protein *A *and protein *C *can be detected.

In this paper, we apply the transitivity concept to peptide fragments instead of the protein sequences to obtain local similarities between remotely homologues. Protein *A *and protein *C *share local similarity if there is a peptide fragment *similar *(formal definition of peptide similarity will be discussed in next subsection) to subsequences in protein *A *and protein *C*. Figure 1(b) illustrates the idea, in which protein *A *and *C *are aligned with protein *B1 *and protein *B2 *(*B1 *and *B2 *can be identical, homologous or non-homologous). If there is a peptide fragment shared by both *B1 *and *B2*, the corresponding peptide fragments in protein *A *and protein *C *are inferred as locally similar between protein *A *and protein *C*. The shared peptide may represent a possible sequence variation in evolution. Moreover, if protein *A *and protein *C *are remotely homologous, there is likely more "shared" sequence fragments in different protein *B*'s to characterize their similarity. However, not all such proteins *A *and *C *which share local similarity are homologous. Some local similarities may arise without common ancestry. Short sequences may be similar by chance, and sequences may be similar because both are selected to bind to a particular protein. In order to avoid ambiguity, we define such proteins *A *and *C *which share local similarity as "*related sequences*".

### Construction of the knowledge base *SPKB*

**Figure 2:**
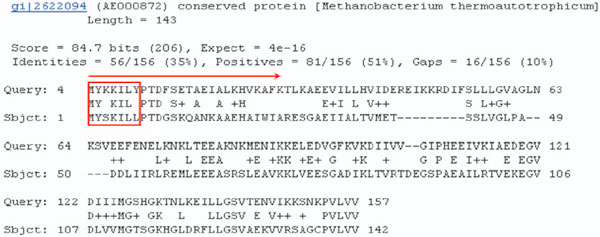
**A real example of HSP found by PSI-BLAST**. We define that MYSKILL (assuming that the window size is 7) is a similar peptide of MYKKILY and we treat it as an extended sequence feature of the query protein. The similarity level of MYSKILL and MYKKILY is 5 since there are five interchangeable residue pairs within that window. We can generate multiple similar peptides from protein gi|2622094 (Sbjct) for the query protein.

Given a dataset of proteins with known localization sites, we construct a knowledge base, called *Similar-Peptide Knowledge Base *(or *SPKB *in short). The dataset used to construct *SPKB *will be described in the Result section. To construct the knowledge base, we first use the native sequence of each protein in the dataset to extract the fixed-length peptide fragments by using a sliding window of length *w*. Each peptide sequence as well as its protein source and the localization site information are stored in *SPKB*. Since the performance of knowledge-based methods relies on the size of the knowledge base, we then perform PSI-BLAST search with parameters *j *= 3, *e *= 0.001 on each protein in the dataset against the NCBInr database to find similar sequences. Since the NCBInr database contains only the protein sequence information, the localization annotation of peptides generated by similar sequences is determined as follows. Specifically, given a query protein sequence *q*, PSI-BLAST would generate a large number of significant local pairwise alignments called *high-scoring segment pairs *(HSPs) between *q *and its similar proteins. An example of an HSP is shown in Figure 2. Statistically significant BLAST hits usually signify sequence homology. We assumed that in an HSP, the *similar peptide sequences *in the counterpart sequence (denoted by "*Sbjct*") represent the possible sequence variations to the corresponding peptide in the query (denoted by "*Query*"), i.e., the protein *q*. We use the same sliding window of length *w *to generate all peptide fragments in each HSP. Two amino acids aligned together in an HSP are said to be *interchangeable *if they have a positive score in the BLOSUM62 (an interchangeable residue pair is represented as an amino acid letter or a plus symbol in an HSP). The number of amino acid pairs being interchangeable within a sliding window represents the *similarity level *of the two peptide fragments. A peptide in *Sbjct *is called a *similar peptide *if it has at least *k *residues interchangeable to those of the corresponding peptide in *Query*. A similar peptide is used to signify local sequence similarity between *Sbjct *and *Query *and thus is assigned the localization annotation of the protein *q*.

Performing PSI-BLAST search for all proteins in the dataset, we can generate a huge number, possibly multi-millions, of similar peptides with localization annotation. Each record in the knowledge base is indexed by a similar peptide, and stores its similar peptide sequences and protein sources (those that are used as query proteins in the PSI-BLAST searches), similarity level and localization site information (inferred from the corresponding protein sources). Note that a similar peptide may occur multiple times in different HSPs of a single PSI-BLAST search result, i.e., derived from different similar proteins found in the PSI-BLAST search. We cluster them together and store the frequency in the peptide record. Table 1 shows a record of the similar peptide MYSKILL (assuming that the window size is 7), which is generated by performing PSI-BLAST search on the three proteins (*A*, *B*, and *C*) with known localization sites, respectively. The frequencies of MYSKILL in the PSI-BLAST search results of proteins *A*, *B*, and *C *are 21, 12, and 17, respectively. The localization site information is inherited from the three protein sources.

**Table 1:** A similar peptide example.

Similar Peptide: MYSKILL				
**Protein Source**	**Localization Sites**	**Native Peptide Sequence**	**Similarity Level**	**Frequency**

*A*	Cytoplasm	MYKKILY	5	21
*B*	Nuclear	MYSSIIL	4	12
*C*	Cytoplasm Extracellular	MYSSILY	5	17

Three protein sources with known localization sites contain peptides that are aligned and similar to the peptide MYSKILL in their HSPs. The similarity level indicates the number of amino acid pairs that are interchangeable between the native peptide sequence and the similar peptide sequence. The frequency represents the number of occurrences they are aligned in HSPs.

### KnowPred_site_: a localization prediction method using *SPKB*

**Figure 3:**
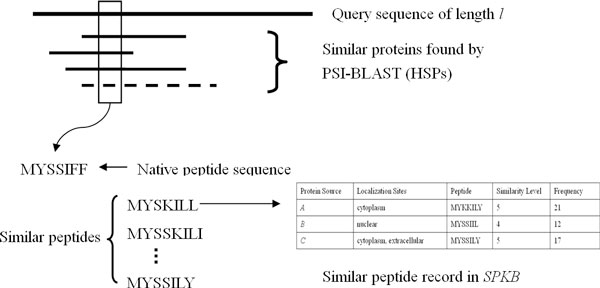
**The main algorithm of KnowPred_site_**.

The main idea of KnowPred_site _is illustrated in Figure 3. Given a target protein *t*, whose localization annotation is unknown and to be predicted, we perform PSI-BLAST search and use the same procedure as described in the last subsection for knowledge base construction to generate all similar peptides of *t *and their frequencies from its native sequence and HSPs. Each similar peptide *hp *is then matched against *SPKB*, and the peptide record with index *hp *is called a *hit*. For each hit, we calculate two types of scores associated with each localization site *i*: the voting score *s*_*i *_and the confidence score *CS*(*i*). The calculation of the voting score *s*_*i *_is as follows: Let *f *denote the frequency of *hp *found in all *t*'s HSPs. For each record in *SPKB*, we calculate the score *w*_*i *_associated with each localization site by summing up the frequencies of the similar peptides that contain the specific site. For example, for the peptide record MYSKILL shown in Table 1, the score of cytoplasm is 38 (21+17; since protein source *A *and *C *are both localized into cytoplasm), and those of nuclear and extracellular are 12 and 17, respectively. Then the voting score *s*_*i *_is defined as *f *multiplied by (*w*_*i*_/total frequencies in that record). For example, if MYSKILL is a similar peptide of *t *and its frequency is 10 in *t's *HSPs, then the voting scores of cytoplasm, nuclear, and extracellular are 7.6 (=10 × 38/50), 2.4 (=10 × 12/50), and 3.4 (=10 × 17/50), respectively, while those of other localization sites are all 0.

The localization site prediction of the protein *t *is determined by the confidence score *CS*(*i*), which is the total voting score aggregated from all hit records. Finally, each *CS*(*i*) is divided by the summation of all frequencies *f *of all *t'*s hits and then multiplied by 100 to normalize the confidence score in the range of 0 and 100. KnowPred_site _predicts *t *being localized into the site with the highest confidence score for single-localized proteins or into the sites with the two highest confidence scores for multi-localized proteins (All multi-localized proteins in ngLOC dataset have two localization sites).

To differentiate single-localized proteins from those that are multi-localized, we followed King and Guda's method [23] to calculate the multi-localized confidence score (*MLCS*) associated with a protein *t*, which gives a relative measure of the likelihood that the protein *t *is multi-localized. It is derived from the two highest confidence scores (denoted as *CS*_1 _and *CS*_2_) and is defined as follows:

and *MLCS*(*t*) is bounded by 100, i.e., when the calculated *MLCS*(*t*) is over 100, it is assigned 100.

### BLAST-hit prediction method

Since BLAST is the most popular method for sequence comparison, we implemented a simple prediction method based on the BLAST search result. Given a dataset of proteins with known localization site(s), to predict the localization site(s) of a test protein *t *we first perform the BLAST search against the dataset and then assign the localization annotations of the best BLAST hit to the protein *t*. If there is no hit at the e-value cutoff 0.001, no annotation will be assigned to the protein *t*. As reported by Jones and Swindells, the e-value of 0.001 generally produces a safe searching [28]. The performance of BLAST-based prediction method is usually treated as the baseline to compare with those of other methods [29].

### Evaluation measure

The performance is estimated using the following measurements. To assess the performance in each localization site, precision, accuracy and Matthew's correlation coefficient (*MCC*) are calculated by Equations (1) to (3), respectively. The overall accuracy is defined in Equation (4).

where *TP*_*i*_, *TN*_*i*_, *FP*_*i*_, *FN*_*i*_, and *N*_*i *_are, respectively, the number of true positives, true negatives, false positives, false negatives, and proteins in localization site *i*. *MCC*, which considers both under- and over-predictions, provides a complementary measure of the predictive performance, where *MCC *= 1 indicates a perfect prediction, *MCC *= 0 indicates a completely random assignment, and *MCC *= -1 indicates a perfectly reverse correlation.

## Results

KnowPred_site _was implemented as a parallel program under the Linux environment. It was implemented using C++ and MPICH library. We used the ngLOC dataset [23] to construct the knowledge base and test the performance of KnowPred_site_. The dataset is compiled from 1923 different species and contains 28056 protein sequences (listed in Additional file 1), including 25887 single localized proteins and 2169 multi-localized proteins. There are ten different subcellular locations among these proteins, which are Cytoplasm (CYT), Cytoskeleton (CSK), Endoplasmic Reticulum (END), Extracellular (EXC), Golgi Apparatus (GOL), Lysosome (LYS), Mitochondria (MIT), Nuclear (NUC), Plasma Membrane (PLA), and Perixosome (POX).

We conducted two types of experiment on the dataset. First, in order to take advantages of local similarities from as many proteins as possible, we conducted the leave-one-out cross validation experiment to determine the parameters and to evaluate the performance of KnowPred_site_. In this experiment, each protein was in turn used as the test protein and the remaining 28055 proteins were used to construct the knowledge base. Second, we compared the performance of KnowPred_site _with existing methods. Since the dataset is from ngLOC and ngLOC has been shown to be better than PSORT [30], pTARGET [31] and PLOC [32] using the same dataset, we directly compare KnowPred_site _against ngLOC using ten-fold cross validation. In this experiment, all proteins were partitioned into 10 subsets, and each subset was in turn used as the test set and the remaining nine subsets were used to construct the knowledge base.

### Determining window size w and similarity threshold *k *for KnowPred_site_

KnowPred_site _aims to utilize the localization annotations of similar peptides. The determination of similar relations, which depends on the window size *w *and the threshold of similarity level *k*, can affect the performance of KnowPred_site_. Using a smaller *w*, similar peptides have a higher probability to be hit against the knowledge base; however, shorter peptide sequences are likely to appear in many unrelated proteins. Given a fixed *w*, there is also a trade-off in choosing the threshold of similarity level *k*. A smaller *k *produces looser similarity relations, which leads to extracting more, but less reliable, similar peptides. To make an appropriate selection of *w *and *k*, we conducted a leave-one-out cross validation experiments on only the single-localized proteins in the ngLOC dataset for *w *ranging from 3 to 11 and *k *ranging from 0 to *w*.

**Figure 4:**
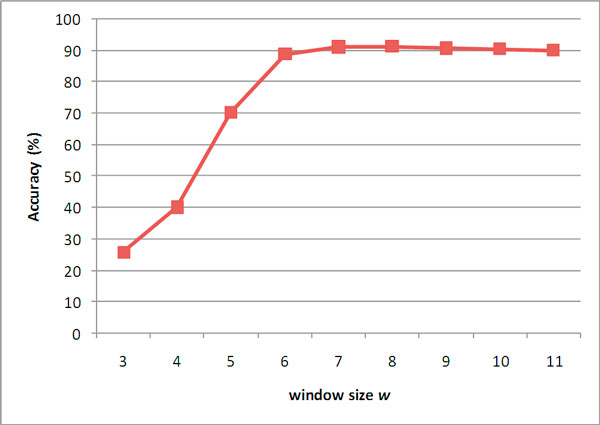
**The overall accuracies of KnowPred_site _using different size of similar peptide length**.

Figure 4 shows the overall accuracies of KnowPred_site _using different window size *w *with fixed similarity threshold (*k *= 0). It shows that the appropriate window size is 7 or 8. Then we further investigate the performance using different thresholds of similarity levels. Table 2 shows the overall accuracies ranging from 90.9% to 92.0% for all combinations of window sizes (*w *= 7, 8) and similarity thresholds. According to the experiment results, we chose the combination of *w *= 7 and *k *= 6 for the following experiments since they provided the best accuracy 92.0%.

**Table 2:** The overall accuracies using different thresholds of similarity levels for window size 7 and 8.

Similarity Level Threshold *k*	0	1	2	3	4	5	6	7	8
*Overall Accuracy *(%) w = 7	91.2	91.2	91.3	91.4	91.5	91.8	92.0	91.6	-
*Overall Accuracy *(%) w = 8	91.4	91.4	91.4	91.4	91.4	91.5	91.6	91.7	90.9

The combination of w = 7 and k = 6 provides the best accuracy. Some results are shown to have identical overall accuracies due to the rounding off to the first decimal place.

### Prediction performance of KnowPred_site_

After the best parameters have been determined, we conducted a ten-fold cross validation experiment on the entire dataset to compare KnowPred_site _with ngLOC and Blast-hit prediction. We used the top *N *accuracy for evaluation, where *N *ranges from 1 to 4. A protein is considered to be correctly predicted when the real localization site(s) rank among the top *N *of the predicted sites. (Top 1 accuracy is simply the *Accuracy *defined in Equation (4).) Notably, for multi-localized proteins, the accuracy is measured in two ways: first, at least one site correctly predicted and second, both sites correctly predicted. Using the first measurement, a true positive is a multi-localized protein with at least one localization site correctly predicted; whereas a true positive using the second measurement is a multi-localized protein with both sites correctly predicted.

The prediction performance of KnowPred_site_, ngLOC, and Blast-hit is summarized in Table 3, in which KnowPred_site _performance is reported with ten-fold cross validation and leave-one-out cross validation as denoted by ^#^KnowPred_site _and *KnowPred_site_, respectively. It is observed that KnowPred_site _outperforms ngLOC and Blast-hit. (The prediction results of single- and multi-localized proteins by KnowPred_site _can be found in Additional file 2 to Additional file 5. Additional file 2 lists the prediction results for single-localized proteins using leave-one-out cross validation; Additional file 3 lists the prediction results for single-localized proteins using ten-fold cross validation; Additional file 4 lists the prediction results for multi-localized proteins using leave-one-out cross validation; Additional file 5 lists the prediction results for multi-localized proteins using ten-fold cross validation.)

**Table 3:** Prediction performance of KnowPred_site_, ngLOC, and Blast-hit

*Overall Accuracy *(%)	Methods	Top 1	Top 2	Top 3	Top 4
Single-localized	*KnowPred_site_	92.0	95.7	96.8	98.1
	^#^KnowPred_site_	91.7	95.4	96.6	97.9
	ngLOC	88.8	92.2	94.5	96.3
	Blast-hit	86.0	-	-	-

Multi-localized (at least 1 correct)	*KnowPred_site_	90.8	96.4	98.2	98.9
	^#^KnowPred_site_	90.1	96.1	98.1	98.9
	ngLOC	81.9	92.0	96.1	97.4
	Blast-hit	78.8	-	-	-

Multi-localized (both correct)	*KnowPred_site_		74.3	83.3	88.7
	^#^KnowPred_site_		72.1	82.2	87.5
	ngLOC		59.7	73.8	83.2
	Blast-hit		45.7	-	-

*KnowPredsite represents the experiment result using leave-one-out cross validation; #KnowPredsite represents the experiment result using 10-fold cross validation.

For single-localized proteins, the overall accuracies of KnowPred_site _are from 91.7 to 98.1 when the correct prediction is considered within the top 1 to top 4 most probable sites. Those of ngLOC are from 88.8% to 96.3%. The accuracy of Blast-hit is 86.0%, which means 86.0% of single-localized proteins could be correctly predicted by BLAST searches. It is noteworthy that 2114 sequences among all single-localized proteins failed to find significant similar proteins by Blast-hit method; however, 58.8% of them were correctly predicted by KnowPred_site_. It shows that the local similarity helps identify related sequences for subcellular localization prediction.

The experiment result shows that KnowPred_site _has much higher accuracy on multi-localized proteins than the other methods. Using the first accuracy measurement, i.e., at least one site correctly predicted, KnowPred_site _achieves more than 90% of the top 1 accuracy, which is higher than ngLOC by 8.2%. Using the tighter second accuracy measurement, KnowPred_site _achieves 72.1% of the top 2 accuracy, which is higher than ngLOC by 12.4%. Further observing the top N accuracy, we find that KnowPred_site _is more able to narrow down the number of false positives than ngLOC.

The top 1 and top 2 accuracies of the Blast-hit method are 78.8% and 45.7% for the two accuracy measurements. Notably, 318 proteins among all multi-localized proteins failed to find any significant Blast hit; however, 73.3% and 49.7% of them were correctly predicted by KnowPred_site _using the two accuracy measurements, respectively.

### Site-specific prediction performance

In contrast to the overall accuracy of the dataset reported in Table 3, we further analyze the prediction performance on each of the 10 distinct localization sites. The results are summarized in Table 4. Among the 10 localization sites, the precision ranges from 75.7% to 98.5% and the accuracy_*i *_ranges from 52.0% to 96.4%. It is observed that higher occurrence of the localization site, e.g., EXC (29.1%) and PLA (25.2%), leads to better prediction, e.g., the precision and accuracy on EXC are 98.5% and 93.9%, respectively. Low occurrence of the localization site can deteriorate prediction, for example, CSK (1%) and GOL (1.1%) have MCC_i_ of 0.645 and 0.746, respectively. However, if the similar peptide records of a site have higher specificity, prediction performance can be good despite low occurrence. For example, the precision and accuracy on LYS (0.6%) and POX (0.8%) are 87.2% and 81.9%, and 87.3% and 85.1%, respectively. Furthermore, it is noteworthy that although CYT represents 11.1% of the dataset, its MCC_i_ is 0.774, much lower than other highly occurring sites. Its low MCC_i_ is due to low precision since KnowPredsite yields more false positives for CYT. High false positives usually occur when the similar peptide records of a site have lower specificity and higher diversity. As a result, proteins of other localization sites are misclassified as CYT.

**Table 4:** Prediction performance of KnowPred_site_ for each site using precision, accuracy, and MCC.

Site *i*	Occurrence in the dataset (%)	*Precision *(%)	*Accuracy*_*i *_(%)	***MCC***_*i*_
CYT	11.1	75.7	84.4	0.774
CSK	1.0	81.1	52.0	0.645
END	3.6	92.9	84.1	0.88
EXC	29.1	98.5	93.9	0.946
GOL	1.1	79.1	70.9	0.746
LYS	0.6	87.2	81.9	0.844
MIT	9.4	96.7	86.9	0.907
NUC	18.0	87.3	93.8	0.884
PLA	25.2	94.4	96.4	0.938
POX	0.8	87.3	85.1	0.861

**Figure 5:**
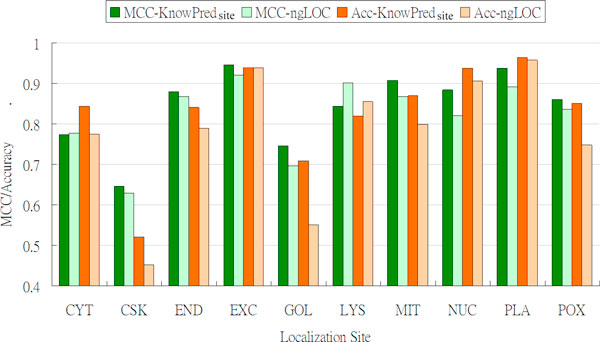
**Matthew's correlation coefficient (*MCC*) and accuracy comparison between KnowPred_site _and ngLOC**.

Figure 5 shows the site-specific comparison between KnowPred_site _and ngLOC in terms of accuracy and MCC. KnowPred_site _outperforms ngLOC in eight localization sites (CSK, END, EXC, GOL, MIT, NUC, PLA, POX) in terms of MCC. The two sites where ngLOC performs better are CYT (0.777 for ngLOC and 0.774 for KnowPred_site_) and LYS (0.902 for ngLOC and 0.844 for KnowPred_site_). In terms of accuracy, KnowPred_site _outperforms ngLOC in all sites except for LYS (representing around 0.6% of the whole dataset), where ngLOC and KnowPred_site _yields 85.5% and 81.9% of accuracy, respectively.

### Evaluation of the multi-localized confidence score (MLCS)

A significant number of eukaryotic proteins are known to be localized into multiple subcellular organelles; therefore, it is important to differentiate single-localized proteins from multi-localized proteins. We used the entire ngLOC dataset to compare different MLCS thresholds on the correct distinction between single-localized and multi-localized proteins. Specifically, we used the portions of true positives in the multi-localized proteins and true negatives in the single-localized proteins as the performance measures. A true positive represents a multi-localized protein whose MLCS is above the threshold and a true negative represents a single-localized protein whose MLCS is below the threshold.

**Figure 6:**
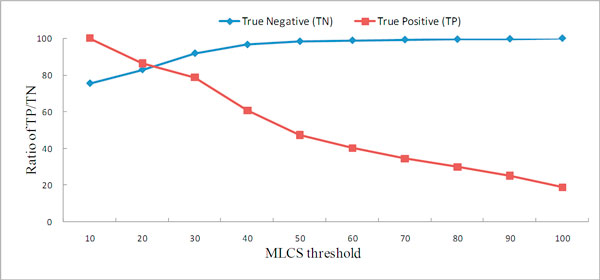
**MLCS analysis**. A true positive represents a multi-localized protein whose MLCS is above the threshold and a true negative represents a single-localized protein whose MLCS is below the threshold. We compare the ratio of true positives/true negatives to the total number of multi-/single-localized proteins.

We illustrate the cumulative percentages of true positive and true negative versus the MLCS threshold in Figure 6, which shows that the true negative curve is increasing along the MLCS axis whereas the true positive curve is decreasing. If the MLCS threshold is set to be 40, 60.7% of multi-localized proteins are true positives and 96.5% of single-localized proteins are true negatives. It shows that 60.7% of multi-localized proteins obtained MLCS of 40 or better, whereas only 3.5% of single-localized proteins within this range. If the MLCS threshold is set to be 20, 86.3% of multi-localized proteins are true positives and 82.8% of single-localized proteins are true negatives. In ngLOC, the best result shows that 76% of multi-localized proteins belong to true positives and 81% of single-localized proteins belong to true negatives when 40 of MLCS threshold is applied. The result shows that KnowPred_site _better differentiate multi-localized proteins from those that are single-localized.

## Discussion

Unlike most machine learning methods that the parameters of the prediction models are not biologically interpretable, the prediction result of KnowPred_site _is interpretable and the prediction process is transparent and traceable. To predict the localization sites of a protein, KnowPred_site_ shows the template sequences and their associated contributive confidence scores for a query protein. Such information is useful for interpretation of the prediction results. In this section, we select the four sequences EF1A2_RABIT, RASH_HUMAN, MCA3_MOUSE, and CFDP2_BOVIN from the ngLOC dataset, to demonstrate the interpretation of KnowPred_site _prediction results.

The prediction result of each of the first three proteins and its template sequences extracted from the knowledge base used for prediction are shown in Table 5, 6, 7, respectively. In each table, the prediction result shows the MLCS and the confidence score of each localization site that the query protein would be localized into. Moreover, the template proteins which are used to vote for the localization sites are shown in each table. We only list the top eight template proteins which contribute most to the confidence scores of the query sequence. For each template sequence, its contribution to confidence score of each localization site and the sequence identity to the query protein calculated by ClustalW (denoted by SI) are shown.

**Table 5:** Prediction result of EF1A2_RABIT.

Query	CYT	CSK	END	EXC	GOL	LYS	MIT	NUC*	PLA	POX	MLCS
EF1A2_RABIT	95.45	0	0	1.45	0	0	0.04	2.97	0.05	0	7.40

											

Template	CYT	CSK	END	EXC	GOL	LYS	MIT	NUC	PLA	POX	SI

EF1A2_RAT	0	0	0	0	0	0	0	2.94	0	0	99.78
EF1A_CHICK	2.77	0	0	0	0	0	0	0	0	0	92.22
EF1A1_HUMAN	2.75	0	0	0	0	0	0	0	0	0	92.22
EF1A1_RAT	2.75	0	0	0	0	0	0	0	0	0	92.22
EF1A0_XENLA	2.69	0	0	0	0	0	0	0	0	0	90.06
EF1A_BRARE	2.64	0	0	0	0	0	0	0	0	0	90.06
EF1A2_XENLA	2.64	0	0	0	0	0	0	0	0	0	88.79
EF1A3_XENLA	2.60	0	0	0	0	0	0	0	0	0	88.55

*: correct answer; SI: sequence identity.

**Table 6:** Prediction result of RASH_HUMAN.

Query	CYT*	CSK	END	EXC	GOL*	LYS	MIT	NUC	PLA	POX	MLCS
RASH_HUMAN	18.95	0.06	0.09	0.09	13.74	0.04	0.24	0.25	83.61	0	36.24

											

Template	CYT	CSK	END	EXC	GOL	LYS	MIT	NUC	PLA	POX	SI

RASK_HUMAN	0	0	0	0	0	0	0	0	13.88	0	86.32
RASK_MOUSE	0	0	0	0	0	0	0	0	13.81	0	86.32
RASN_HUMAN	13.19	0	0	0	13.19	0	0	0	0	0	85.19
LET60_CAEEL	0	0	0	0	0	0	0	0	10.55	0	74.07
RAS3_RHIRA	0	0	0	0	0	0	0	0	5.05	0	57.07
RAS1_RHIRA	0	0	0	0	0	0	0	0	4.88	0	58.62
RAS2_RHIRA	0	0	0	0	0	0	0	0	4.33	0	35.20
RAS_LIMLI	0	0	0	0	0	0	0	0	4.15	0	46.03

*: correct answer; SI: sequence identity.

**Table 7:** Prediction result of MCA3_MOUSE. Templates marked with '+' are those that have the same localization annotation with the query protein.

Query	CYT*	CSK	END	EXC	GOL	LYS	MIT	NUC*	PLA	POX	MLCS
MCA3_MOUSE	95.46	0.3	0.27	0.36	0.2	0.01	1.13	93.59	1.82	0.22	100

											

Template	CYT	CSK	END	EXC	GOL	LYS	MIT	NUC	PLA	POX	SI

MCA3_HUMAN^+^	89.16	0	0	0	0	0	0	89.16	0	0	88.51
EF1G1_YEAST^+^	2.74	0	0	0	0	0	0	2.47	0	0	8.67
EF1G2_YEAST	0.49	0	0	0	0	0	0.49	0	0	0	8.50
GSTA_PLEPL	0.35	0	0	0	0	0	0	0	0	0	15.86
SYEC_YEAST	0.16	0	0	0	0	0	0	0	0	0	3.86
CCNA1_MOUSE	0	0.15	0	0	0	0	0	0	0	0	7.36
NU155_RAT^+^	0.14	0	0	0	0	0	0	0.14	0	0	3.17
GCYB2_HUMAN	0.14	0	0	0	0	0	0	0	0	0	4.86

*: correct answer; SI: sequence identity.

In the example of EF1A2_RABIT shown in Table 5, KnowPred_site _predicts it being single-localized at cytoplasm (CYT) since MLCS is very low (7.40) and CYT has the highest confidence score. However, the localization site of EF1A2_RABIT reported in the ngLOC dataset is nuclear (NUC). Examining the eight template proteins, we find that they all have high sequence identities with EF1A2_RABIT and most of them are localized into CYT except EF1A2_RAT localized into NUC. According to the Gene Ontology annotation, it is localized into CYT and NUC, which are the two sites with the highest confidence scores in KnowPred_site_'s prediction.

In the example of RASH_HUMAN shown in Table 6, KnowPred_site _predicts RASH_HUMAN being localized into plasma membrane (PLA) and cytoplasm (CYT). However, the correct localization site is cytoplasm and Golgi apparatus (GOL). Referring to the prediction result, the confidence score of PLA is much higher than those of CYT and GOL. It is also observed that most of the template proteins are localized into PLA. According to the annotation in Gene Ontology and SwissProt, RASH_HUMAN is localized into PLA and GOL, and the template protein, RASN_HUMAN, is also localized into PLA and GOL. If applying the new annotation data, KnowPred_site _can predict RASH_HUMAN correctly.

As for MCA3_MOUSE shown in Table 7, KnowPred_site _predicts its MLCS 100 and it being localized into cytoplasm (CYT) and nuclear (NUC) correctly. Examining the template proteins, we observe that KnowPred_site _identifies some related proteins, i.e., which have the same localization with the query protein. EF1G1_YEAST and NU155_RAT, even though they share very low sequence identity 8.67% and 3.17%, respectively, with the query protein. Notably, the two template proteins rank second and seventh, respectively, among all template proteins. Furthermore, though GSTA_PLEPL has higher sequence identity (15.86%) with the query protein than EF1G1_YEAST, the confidence score contributed by EF1G1_YEAST is much higher than that by GSTA_PLEPL (2.74 vs. 0.35). It shows that the contributive confidence score is not necessary to be positively correlated with the sequence identity when template sequences are dissimilar with the query sequence. In this example, EF1G1_YEAST shares more local similarities (peptide fragments) with the query protein than GSTA_PLEPL does. If MCA3_HUMAN, the one that shares 88.51% sequence identity with the query protein, is taken out from the template pool, KnowPred_site _can still predict correctly for protein MCA3_MOUSE.

For the multi-localized proteins, there are 318 proteins unable to find similar sequences by the Blast-hit method. However, the localization sites of around half of them can be correctly predicted by KnowPred_site_. We randomly choose an example, CFDP2_BOVIN, to demonstrate the KnowPred_site_'s capability of identifying related sequences from the template pool. The two highest confidence scores of CFDP2_BOVIN are 32.07 (CYT) and 41.18 (NUC). Among the top 100 templates (ranked by the contribution to the confidence scores), 12 of them are localized into CYT and NUC, 18 are localized into CYT only, and 32 are localized into NUC only. Their sequence identities against CFDP2_BOVIN are very low, ranging from 3.47% to 13.8%. The result suggests that local similarity captured by our method is beneficial for PSL prediction when global sequence similarity is very low.

## Conclusion

In this paper, we propose a highly accurate subcellular localization prediction method for single- and multi-localized proteins, called KnowPred_site_, which is based on a knowledge base instead of frequently used machine learning approaches. The knowledge base, called *SPKB*, is constructed from a given dataset of proteins with known localization site annotation to capture local similarity between proteins so that related proteins with the same localization can be identified. Using these related proteins obtained form the knowledge base, the localization site of a query protein can be better predicted.

We used the ngLOC dataset to evaluate the performance of KnowPred_site_. The dataset consists of 25887 single-localized proteins and 2169 multi-localized proteins of ten subcellular proteomes from 1923 species. In order to compare KnowPred_site _with ngLOC and the baseline Blast-hit method, we performed ten-fold cross validation on the dataset. The experiment results show that KnowPred_site _achieves higher prediction accuracy than ngLOC and Blast-hit. Particularly, on multi-localized sequences KnowPred_site _outperformed ngLOC by 8.2% in accuracy when a protein is correctly predicted if at least one site is correctly identified and by 12.4% in accuracy when a protein is correctly predicted if both sites are correctly identified.

A major advantage of knowledge base approaches is that the prediction process is transparent and interpretable. We can examine the prediction process to see how KnowPred_site _generates the prediction. Furthermore, with close observation from the prediction results in our experiments as described in the Discussion section, we find that KnowPred_site _can efficiently use local similarity to identify related sequences even when their sequence identity is low so as to predict localization site with high accuracy.

When more proteins have known localization sites, most machine learning based methods need to retrain the prediction models, In contrast, KnowPred_site _can be easily improved by incrementally expanding the knowledge base, i.e., adding new peptide records or updating existing records with new protein sources and their localization site information. This feature indicates the expansibility and efficiency in maintaining the KnowPred_site _prediction system.

## Competing interests

The authors declare that they have no competing interests.

## Authors' contributions

Hsin-Nan Lin developed the method, carried out the computational predictions. Ching-Tai Chen and Hsin-Nan Lin were involved in the literature survey, result interpretation, statistical analysis, and manuscript writing. Ting-Yi Sung, Shinn-Ying Ho and Wen-Lian Hsu coordinated the study and revised the manuscript. All authors read and approved the final manuscript.

## Note

Other papers from the meeting have been published as part of *BMC Genomics *Volume 10 Supplement 3, 2009: Eighth International Conference on Bioinformatics (InCoB2009): Computational Biology, available online at http://www.biomedcentral.com/1471-2164/10?issue=S3.

## Supplementary Material

Additional file 1 **ngLOC dataset**. The file contains whole ngLOC dataset, in which the row starts with '>' represents the protein name, the next row represents localization site. The localization site is numbered from 1 to 10, denoting Cytoplasm (CYT), Cytoskeleton (CSK), Endoplasmic Reticulum (END), Extracellular (EXC), Golgi Apparatus (GOL), Lysosome (LYS), Mitochondria (MIT), Nuclear (NUC), Plasma Membrane (PLA), and Perixosome (POX). The ngLOC dataset can be also downloaded via http://bio-cluster.iis.sinica.edu.tw/kbloc/DataSet.htm. Click here for file

Additional file 2 **KnowPred_site _prediction results for single-localized proteins using leave-one-out cross validation**. Each row is a prediction result for a protein sequence. Columns A, and B represent protein name and localization site annotation, respectively. Columns C to L are the confidence scores corresponding to each localization site. Columns N to Q are the Top 1 to Top 4 accuracies. Click here for file

Additional file 3 **KnowPred_site _prediction results for single-localized proteins using ten-fold cross validation**. The columns' definition is the same as that for Additional File 2. Click here for file

Additional file 4 **KnowPred_site _prediction results for multi-localized proteins using leave-one-out cross validation**. Each row is a prediction result for a protein sequence. Columns A to L are the same to Additional File 2. Columns N to Q are the Top 1 to Top 4 accuracies based on the "at least one correct" criterion. Columns S to U are Top 2 to Top 4 accuracies based on the "both correct" criterion. Click here for file

Additional file 5 **KnowPred_site _prediction results for multi-localized proteins using ten-fold cross validation**. The columns' definition is the same as that for Additional file 4. Click here for file
